# Intercalating Ultrathin MoO_3_ Nanobelts into MXene Film with Ultrahigh Volumetric Capacitance and Excellent Deformation for High-Energy-Density Devices

**DOI:** 10.1007/s40820-020-00450-0

**Published:** 2020-05-22

**Authors:** Yuanming Wang, Xue Wang, Xiaolong Li, Rong Liu, Yang Bai, Huanhao Xiao, Yang Liu, Guohui Yuan

**Affiliations:** 1 MIIT Key Laboratory of Critical Materials Technology for New Energy Conversion and Storage, School of Chemistry and Chemical Engineering, Harbin Institute of Technology, No. 92 West Dazhi Street, Harbin, 150001 People’s Republic of China; 2 Ocean College, Hebei Agricultural University, No. 52 east section, Hebei Street, Qinhuangdao, 066000 People’s Republic of China

**Keywords:** MXene, MoO_3_ nanobelts, Hybrid film, Ultrahigh volumetric capacitance, Supercapacitors

## Abstract

The restacking hindrance of MXene films restricts their development for high volumetric energy density of flexible supercapacitors toward applications in miniature, portable, wearable or implantable electronic devices. A valid solution is construction of rational heterojunction to achieve a synergistic property enhancement. The introduction of spacers such as graphene, CNTs, cellulose and the like demonstrates limited enhancement in rate capability. The combination of currently reported pseudocapacitive materials and MXene tends to express the potential capacitance of pseudocapacitive materials rather than MXene, leading to low volumetric capacitance. Therefore, it is necessary to exploit more ideal candidate materials to couple with MXene for fully expressing both potentials. Herein, for the first time, high electrochemically active materials of ultrathin MoO_3_ nanobelts are intercalated into MXene films. In the composites, MoO_3_ nanobelts not only act as pillaring components to prevent restacking of MXene nanosheets for fully expressing the MXene pseudocapacitance in acidic environment but also provide considerable pseudocapacitive contribution. As a result, the optimal M/MoO_3_ electrode not only achieves a breakthrough in volumetric capacitance (1817 F cm^−3^ and 545 F g^−1^), but also maintains good rate capability and excellent flexibility. Moreover, the corresponding symmetric supercapacitor likewise shows a remarkable energy density of 44.6 Wh L^−1^ (13.4 Wh kg^−1^), rendering the flexible electrode a promising candidate for application in high-energy-density energy storage devices.

**Electronic supplementary material:**

The online version of this article (10.1007/s40820-020-00450-0) contains supplementary material, which is available to authorized users.

## Highlights

- All-pseudocapacitive and highly deformable MXene hybrid film is successfully fabricated by a facile and efficient vacuum-assisted filtration of ultrathin MoO_3_ nanobelts and delaminated MXene nanosheets.
- The optimal M/MoO_3_ hybrid electrode delivers an ultrahigh volumetric capacitance of 1817 F cm^−3^ (545 F g^−1^), which exceeds large majority of previously reported MXene-based flexible electrodes.
- The symmetric supercapacitor presents excellent energy density of 44.6 Wh L^−1^ (13.4 Wh kg^−1^), indicating the electrode promising in achieving high-energy-density devices.

**Electronic supplementary material:**

The online version of this article (10.1007/s40820-020-00450-0) contains supplementary material, which is available to authorized users.

## Graphical abstract

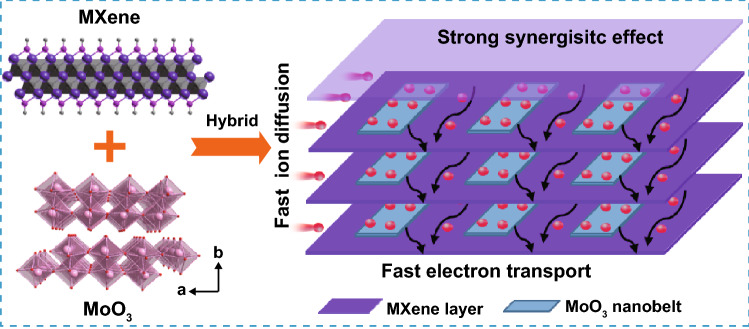

## Introduction

Recently, MXene (Ti_3_C_2_T_*x*_) has attracted great attention in the field of electrochemical energy storage, especially supercapacitors, predominantly due to their unique physical and chemical properties [1–3]. MXene has the similar planar geometry structure as the graphene, while the features of large specific surface area, few atomic thickness and unwrinkled flat surface render MXene materials be easily proceeded into thin film with robust mechanical strength and excellent flexibility [4–6]. More importantly, benefiting from MXene abundant and modifiable surface-terminating moieties, the self-supporting MXene films fabricated by simple vacuum filtration of delaminated MXene nanosheet colloid can directly serve as the flexible supercapacitor electrodes because of not only their flexibility and mechanical stability but also remarkable volumetric capacitance [7–9]. This brings great opportunities to develop next-generation high volumetric energy density of flexible supercapacitors for applications in electronic devices toward the development trend of miniaturization, portability, wearability and biomedical implantation. However, similar to other 2D nanomaterials, MXene nanosheets are easy to aggregate and restack during electrode fabrication process, which seriously impedes rapid diffusion of electrolyte ions and influences the full use of active surface of the electrodes, thus resulting in limited specific volumetric capacitance especially at high rates [10, 11].

A valid route to solve the restacking hindrance of MXene materials is construction of heterojunction by taking both advantages of selected target materials which can provide one or few functions such as good electrical conductivity, abundant electrochemically active sites as well as interlayered pillaring component and MXene materials to achieve a synergistic property enhancement. For example, by assembling graphene and MXene into stacked 2D heterojunction, the rate capability of MXene-based hybrid electrode was enhanced to some extent due to the metallic electrical conductivity of graphene and larger 2D open structure of MXene [12]. Besides, CNTs [13], cellulose [14], PVA (polyvinyl alcohol) [15] and the like have also been employed to fabricate hybrid electrodes with MXene. However, these space materials are low active or inactive in energy storage, and the capacitive performance of electrodes demonstrates limited enhancement. Moreover, based on the surface chemical properties, MXene can provide a particularly suitable 2D building platform for some pseudocapacitive materials such as transition metal oxide (TMO) and layered double hydroxide (LDH) [16, 17]. Nevertheless, arbitrarily grown arrays of plates or rods on MXene substrate face the problems of insufficient mutual contact, inefficient utilization of active materials and failed film forming, resulting in sacrificing electrode flexibility and volumetric capacitance. Furthermore, it has been acknowledged that MXene materials exhibit highest capacity in the acidic electrolytes, while many efforts around combination of pseudocapacitive materials and MXene are conducted in the neutral or alkaline electrolytes because of acidic erosion for many active materials [18, 19]. The mismatch of electrolyte would bring about low capacitive contribution of MXene in the electrodes. In other words, MXene predominantly provides the conductive substrate for hybrid electrodes, finally leading to low specific volumetric capacitance and difficult to acquire a breakthrough of 1500 F cm^−3^ reported in pristine MXene hydrogel film [3]. Therefore, to obtain high-performance electrode, it is necessary to exploit more ideal candidate materials to couple with MXene for fully expressing both potentials.

Pseudocapacitive material of MoO_3_ nanobelts shows promising potential for MXene films including simple preparation process, mechanical stability, high electrochemical reaction activity and, more importantly, high pseudocapacitance in acidic environment [20, 21]. Herein, for the first time, M/MoO_3_ hybrid films are fabricated by simple blending of MXene nanosheet suspension and MoO_3_ nanobelt dispersion and then experiencing vacuum-assisted filtration process. In the composites, the synthesized MoO_3_ nanobelts are ultrathin (~ 16 nm), which is beneficial for the sufficient contact with the conductive MXene substrates to reduce intrinsic resistance and exposing more electrochemically active sites for high capacitive behavior. Meanwhile, MoO_3_ nanobelts serve as the effective interlayers between MXene nanosheets to prevent MXene restacking and render the capacitance of MXene be fully expressed. As a result, the M/MoO_3_ hybrid electrode with MoO_3_ mass fraction of 20% exhibits an ultrahigh volumetric capacitance up to 1817 F cm^−3^ (545 F g^−1^) at a scan rate of 3 mV s^−1^ in 1 M H_2_SO_4_ electrolyte, which exceeds large majority of previously reported MXene-based electrode materials and maintains good rate capability (773 F cm^−3^ at 200 mV s^−1^). Benefiting from the ultrathin feature of both MXene and MoO_3_, the hybrid film presents high deformation (bendable, twistable and even foldable). Moreover, symmetric supercapacitor can yield a volumetric energy density of 44.6 Wh L^−1^ (13.4 Wh kg^−1^), which belongs to the excellent performance in comparison with previously reported MXene-based symmetric supercapacitors. The work provides a simple and feasible strategy to design and fabricate advanced MXene-based flexible electrode with both high electrochemical performance and good flexibility, showing great potential for application in future flexible and portable electronics.

## Experimental Section

### Preparation of Delaminated MXene Nanosheets

Briefly, 3.2 g of LiF (Aladdin, 99%) was dissolved into 40 mL of 9 M HCl aqueous solution and the solution was stirred with a magnetic Teflon stir bar for 5 min to dissolve the salt. Then 2 g of Ti_3_AlC_2_ powders were slowly added to the above mixing solution and the reaction was kept for 48 h at 35 °C to etch the Al atoms in the Ti_3_AlC_2_ phase. Subsequently, the mixture was washed five times at least by adding deionized water until the pH of the supernatant was close to 7. Delaminated MXene suspension (d-Ti_3_C_2_T_*x*_) was prepared by adding deionized water to black sediment settled at the bottom of the centrifuge tube along with vigorous hand-shaking delamination process. After centrifugation at 3500 rpm for 1 h, the supernatant with a color of dark green was collected. The concentration of the MXene suspension was determined by decanting a certain known volume of the suspension into a vial and measuring the weight of the vial after drying.

### Preparation of Ultrathin MoO_3_ Nanobelts

0.96 g Mo powder was carefully added into 12.5 mL H_2_O_2_ (30%) with vigorously stirring for about 1 h in an ice bath until the solution became light yellow. Then 10 g polyethylene glycol (PEG) was added into the above solution with continuously stirring for another 1 h. After this, the solution was transferred to a 30-mL Teflon-lined stainless steel autoclave to undergo a hydrothermal reaction at 150 °C for 12 h. When the mixture cooled down, the product was filtered and washed alternately three times with ethyl alcohol and deionized water. Finally, MoO_3_ nanobelts were dispersed in a known volume of deionized water to obtain the MoO_3_ nanobelts dispersion. The concentration of MoO_3_ nanobelts dispersion was obtained by the same method as that of MXene suspension.

### Preparation of Freestanding M/MoO_3_ (Ti_3_C_2_T_*x*_/MoO_3_) Hybrid Films

Vacuum filtration method was employed to fabricate M/MoO_3_ hybrid films. First, a certain volume of MoO_3_ dispersion was dropwise added into MXene suspension under ultrasonication for 30 min to get a uniformly mixed solution of MXene nanosheets and MoO_3_ nanobelts. Then, the mixed solution was filtered through a filter membrane (0.22 μm pore size). Finally, the freestanding M/MoO_3_ hybrid films were formed by drying at room temperature and peeling off from the filter membrane. M/MoO_3_ hybrid films with different MoO_3_ mass fraction were prepared by increasing the ratio of MoO_3_ to MXene materials. For comparison, the pure MXene film was also fabricated via the same procedure. The mass loading of all as-prepared electrodes was controlled at around 1 mg cm^−2^.

### Characterization

The morphology and microstructure of the as-prepared samples were investigated using field emission scanning electron microscopy (FESEM, Merlin Compact), transmission electron microscopy (TEM, FEI TF30) and atomic force microscope (AFM, Bruker Instruments Dimension Icon). Energy-dispersive spectroscopy (EDX) was performed on an electron microscope at an accelerating voltage of 20 kV. Crystal structures of samples were examined using a powder X-ray diffractometer (XRD) with Cu Kα radiation at a scan rate of 5° min^−1^.

### Electrochemical Measurements

All electrochemical measurements were taken on the CHI660E electrochemical workstation and Neware battery testing system in 1 M H_2_SO_4_ aqueous electrolyte at room temperature. The electrochemical performances of single electrodes were evaluated by the typical three-electrode test configuration, in which self-supporting films were used as work electrode, Ag/AgCl in saturated KCl was the reference electrode and overcapacitive activated carbon served as the counter electrode. The symmetric supercapacitors were assembled with two pieces of identical size of flexible M/MoO_3_-20% films separated by porous nonwoven fabric. The electrochemical impedance spectroscopy (EIS) was conducted within a frequency range from 100 kHz to 0.01 Hz at an amplitude of 5 mV. Cycling stability was measured by repeating the galvanostatic charge/discharge test for 5000 cycles at 30 mA cm^−2^. The gravimetric capacitance was obtained from the discharge portion of cyclic voltammetry (CV) curves through Eq. 1:

1 $$C_{m} = \frac{{\int {I{\text{d}}V} }}{m\upsilon \Delta V}$$

where *I* is the current (mA), *V* is the potential window (V), *m* is the mass of the active materials (mg) and *v* is the scan rate (mV s^−1^), respectively. The volumetric capacitance of film electrodes is calculated according to Eqs. 2 and 3:

2 $$C_{V} = \rho C_{m}$$

3 $$\rho = \frac{m}{Sd}$$

where *S* (cm^−2^) and *d* (cm) are the surface area and thickness of film electrode, respectively. The gravimetric and volumetric energy density (*E*_*m*_, *E*_*v*_) and power density (*P*_*m*_, *P*_*v*_) of symmetric devices are calculated according to Eqs. 4–8:

4 $$E_{m} = \frac{1}{2}CV^{2}$$

5 $$P_{m} = \frac{E}{\Delta t}$$

6 $$\Delta t = \frac{V}{\upsilon }$$

7 $$E_{V} = \rho E_{m}$$

8 $$P_{V} = \rho P_{m}$$

## Results and Discussion

### Film Fabrication and Sample Characterization

**Fig. 1:**
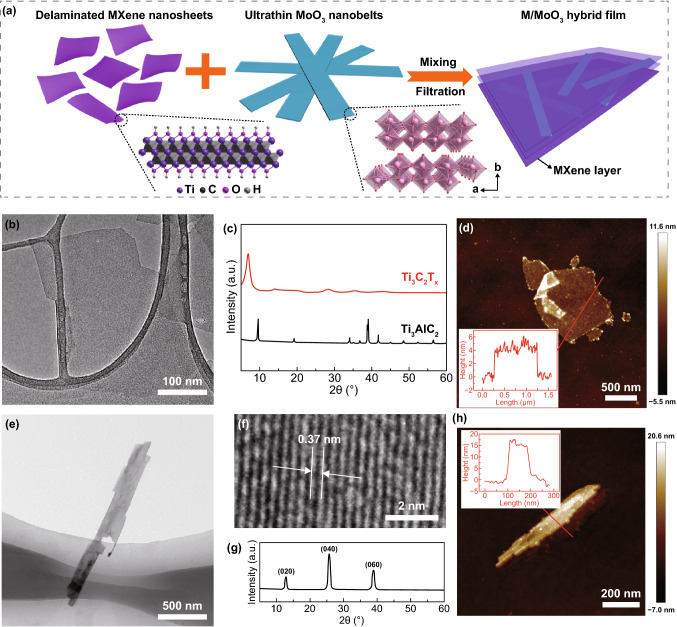
**a** Schematic illustration of the fabrication process of M/MoO_3_ hybrid films, **b** TEM image of delaminated MXene nanosheets, **c** XRD patterns of the MAX (Ti_3_AlC_2_) precursor and the prepared delaminated MXene, **d** AFM image of MXene nanosheets. Inset shows the corresponding height profile along the lines in **d**, **e** TEM and **f** HR-TEM images of MoO_3_ nanobelt, **g** XRD patterns of MoO_3_ nanobelts, **h** AFM image of MoO_3_ nanobelt. Inset shows the corresponding height profile along the lines in **h**

Figure 1a schematically illustrates the preparation process of M/MoO_3_ hybrid films. In a typical synthesis, few-layered Ti_3_C_2_T_*x*_ MXene nanosheet colloidal suspension was synthesized by selectively etching bulk Ti_3_AlC_2_ precursor in LiF and HCl mixed solution, followed by mildly hand-shaking exfoliation. MoO_3_ nanobelts homogeneous dispersion was prepared by a facile one-step hydrothermal process, where Mo powder was served as the Mo source, H_2_O_2_ was the oxidant and PEG (polyethylene glycol) played the role of the crystal growth template for ultrathin feature. Both MXene nanosheet and MoO_3_ nanobelt dispersions show hydrophilic characteristics, which can be highly stable in aqueous media. And the mixture solution including MXene nanosheets and MoO_3_ nanobelts shows good compatibility without any precipitate (Fig. S1). Through vacuum-assisted filtration process, M/MoO_3_ hybrid films with MoO_3_ mass percentage of 10%, 20% and 30% were successfully prepared, which was subsequently denoted as M/MoO_3_-10%, M/MoO_3_-20% and M/MoO_3_-30%, respectively.

TEM image reveals the morphology of the prepared MXene nanosheets which are thin, transparent and possess a lateral dimension of hundreds of nanometers, as illustrated in Fig. 1b. XRD pattern (Fig. 1c) was employed to characterize the structure and phase feature of MAX ceramic powders and delaminated MXene nanosheets. For Ti_3_C_2_T_*x*_ MXene, the (002) diffraction peaks at 6.9° can be observed, which is in consistent with typical MXene with interlayered water molecules as reported in other studies [22]. From AFM characterization (Fig. 1d), the thickness of individual MXene nanosheets is close to 4 nm, demonstrating few-layered MXene is synthesized as single layer of MXene flakes is about 1.5 nm [23]. MoO_3_ morphology was investigated by SEM (Fig. S1) and TEM (Fig. 1e). The MoO_3_ presents ribbonlike characteristics with a width of 100–200 nm and a length of 1–2 μm. The HR-TEM image (Fig. 1f) reveals the nanobelts with an interplanar spacing of 0.37 nm, which is consistent with the (002) *d*-spacing of α-MoO_3_ [24]. Diffraction patterns are shown in Fig. 1g, which can be clearly indexed to be orthorhombic MoO_3_ (JCPDS No. 05-0508) [25, 26]. The strong diffraction peaks of (020), (040) and (060) reveal that the MoO_3_ nanobelts with a highly anisotropic growth own an obviously preferred orientation. Given that α-MoO_3_ is constituted of stacking bilayer sheets of MoO_6_ octahedra with layered structure, the structure is favorable for the infiltration of small electrolyte ions for high capacitive behavior [27–29]. From AFM characterization (Fig. 1h), the height of MoO_3_ nanobelts is only around 16 nm which belongs to the ultrathin size in comparison with other literature reported (~ 100 nm) [24]. The ultrathin feature is not only beneficial for exposing more active sites for high pseudocapacitance, but also sufficient contact with the conductive substrates for fast electron transport.

**Fig. 2:**
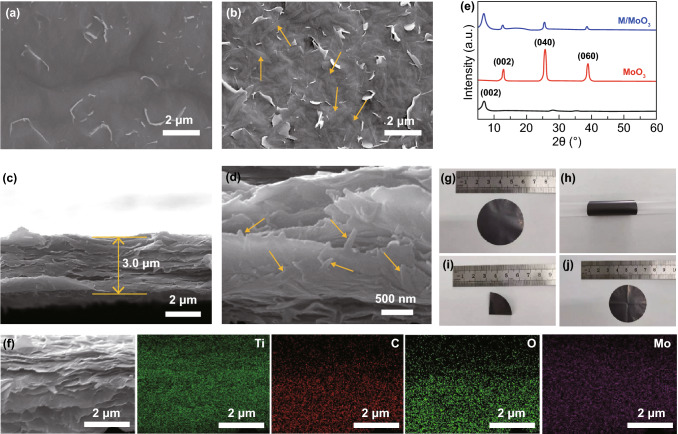
Top view SEM images of **a** pure MXene film and **b** M/MoO_3_-20% hybrid film, **c**, **d** cross-sectional images of M/MoO_3_-20% hybrid film, **e** XRD patterns of MXene film, MoO_3_ nanobelts and M/MoO_3_-20% hybrid film, **f** EDX elemental mapping of Ti, C, O and Mo for the hybrid film, **g**–**j** Optical images of M/MoO_3_ hybrid film at different deformation status, which can be bended, rolled and even folded

Following the material characterizations, the morphology and microstructure of various M/MoO_3_ hybrid films and pure MXene film were investigated by scanning electron microscopy. From the view of top SEM images as shown in Figs. 2a, b and S2a, b, pure MXene film is obviously smoother due to densely stacking of flat MXene nanosheets. After hybridization with MoO_3_, it is found that these nanobelts are scattered in disorder and buried into the MXene substrates which provides continuous conductive networks for improving intrinsic conductivity of MoO_3_ nanobelts. The cross-sectional SEM images (Figs. 2c and S3a, b) indicate that M/MoO_3_ hybrid films maintain a well-aligned lamellar structure. Due to the insertion of nanobelts, the thickness of hybrid films gradually increases with the increasing mass percentage of MoO_3_ because the 2D face-to-face stacking of MXene nanosheets is the denser stacking mode in comparison with the nanobelt–nanosheet stacked structure. However, benefiting from the ultrathin structure of MoO_3_ nanobelts, the thickness increase is considerably limited in favor of achieving high volumetric performance. From the cross-sectional SEM images in high magnification (Fig. 2d), MoO_3_ nanobelts are inserted between the conductive MXene nanolayer, which is considered to be able to reduce the self-stacking problem of MXene nanosheets, thereby enlarging the accessible active surface for energy storage. Meanwhile, MXene could provide binding function for avoiding the active materials loss during the charging/discharging process. In addition, the M/MoO_3_ hybrid film simultaneously exhibits the typical peaks of MXene and MoO_3_ (Fig. 2e), indicating that the addition of MoO_3_ nanobelts does not disturb the MXene stacking order along the c direction as a result of coexistence. The compositional distributions of M/MoO_3_ hybrid film (Fig. 2f) were confirmed by elemental mapping analyses, in which homogeneous distributions of Ti, C, O and Mo elements are clearly showed within the M/MoO_3_ hybrid film. Furthermore, the flexibility of electrodes is the significant assessment norms for flexible energy storage devices. As displayed in Fig. 2g–j, the M/MoO_3_ hybrid film exhibits excellent flexibility and highly deformation, which can be curled around a glass rod and even be folded for many times, while no crack is found in the unfolded film. The tensile strength of hybrid films, as shown in Fig. S4, gradually decreases from 19.1 to 12.5 MPa with the addition of MoO_3_ nanobelts, indicating that the structure of nanobelt–nanosheet stacked structure is relatively looser. Although the tensile strength of hybrid films is lower than that of pure MXene film (22.8 MPa), it still maintains a high level. This also indicates that MXene nanosheets and MoO_3_ nanobelts are assembled tightly together, forming an integrated structure for high mechanical stability and flexibility.

### Electrochemical Performance in a Three-Electrode System

**Fig. 3:**
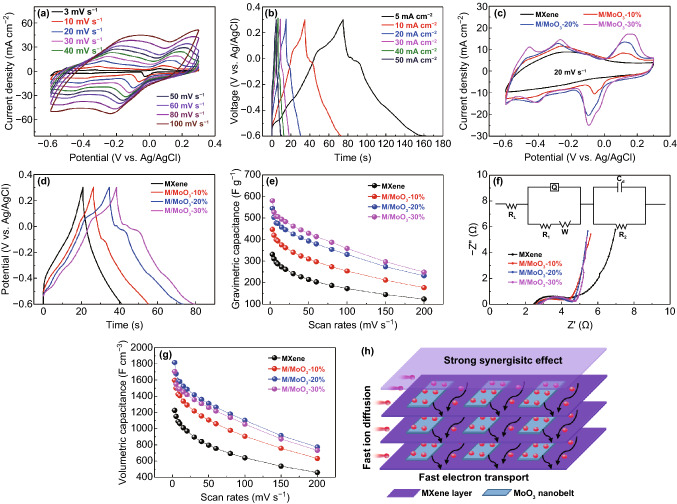
**a** CV curves of the M/MoO_3_-20% electrode at various scan rates, **b** GCD profiles of M/MoO_3_-20% electrode at various current densities, **c** CV curves of pure MXene and hybrid electrodes at a scan rate of 20 mV s^−1^, **d** GCD profiles of pure MXene and hybrid electrodes at a current density of 10 mA cm^−2^, **e** gravimetric specific capacitance of pure MXene and hybrid electrodes calculated from the CV curves of different scan rates, **f** Nyquist plots. Inset shows the equivalent circuit model for the Nyquist plots, **g** corresponding volumetric specific capacitance of various electrodes, **h** proposed schematic diagram of the high capacitive behavior from strong synergistic effect

The electrochemical performance of the as-prepared samples was investigated by using a three-electrode setup in 1 M H_2_SO_4_ aqueous electrolyte in a potential window of − 0.6 to 0.3 V. From CV profiles of the pure MXene (Fig. S5), a couple of broad redox peaks can be clearly observed, demonstrating that the capacitance mainly comes from the pseudocapacitance based on the reversible redox reaction along with the valence state change of the Ti atoms [30]. After the insertion of MoO_3_ nanobelts, as illustrated in Figs. 3a and S6a, c, the CV curves of M/MoO_3_ hybrid electrodes present an obvious difference from those of pure MXene electrode, which involves several pairs of new asymmetric redox peaks at low scan rates, demonstrating MoO_3_ provides pseudocapacitive contribution for the hybrid electrodes. Although at a high scan rate of 100 mV s^−1^, there are obvious redox peaks of MoO_3_ for these hybrid electrodes, indicating fast ion transport for faradaic reaction. Galvanostatic charge–discharge (GCD) profiles of these hybrid electrodes at various current densities are presented in Figs. 3b and S6b, d. All of them present a distortion from ideal triangle shape, where several pairs of charge/discharge plateaus are observed clearly for M/MoO_3_ hybrid electrodes, indicating the pseudocapacitive nature, which is in good according with the results of CV curves. In Fig. 3c, a comparison of CV curves was made between pure MXene electrode and M/MoO_3_ hybrid electrodes at a scan rate of 20 mV s^−1^. In comparison with pure MXene electrode, the M/MoO_3_ hybrid electrodes exhibit a much higher CV integral area and the integral area gradually increases with the increasing mass percentage of MoO_3_, which is also in consistent with the results from GCD profiles (Fig. 3d), reflecting great improvement of the capacitance performance.

To better know the electrochemical behavior of electrodes, the capacitance of the MXene and M/MoO_3_ electrodes were calculated from the CV curves in a wide scan rate range varying from 3 to 200 mV s^−1^, which is shown in Fig. 3e. Obviously, hybrid electrodes exhibit the higher specific capacitance over the whole range of scan rates in comparison with pure MXene electrode. And the specific capacitance of hybrid electrodes gradually rises with the increasing mass percentage of MoO_3_, demonstrating highly electrochemical active materials of MoO_3_ in acidic electrolyte. At a low scan rate of 3 mV s^−1^, the hybrid electrodes deliver a high capacitance of 447, 545 and 580 F g^−1^ corresponding to the samples of M/MoO_3_-10%, M/MoO_3_-20% and M/MoO_3_-30%, respectively, while the pure MXene electrode only yields a capacitance of 331 F g^−1^ at the same scan rate. When the scan rate is increased up to 200 mV s^−1^, the hybrid electrodes still maintain 177 (M/MoO_3_-10%), 232 (M/MoO_3_-20%) and 249 F g^−1^ (M/MoO_3_-30%), considerably higher than that of pure MXene electrode (124 F g^−1^). This indicates that the rate capability of hybrid electrodes does not drop off although large enhancement of capacitance performance in comparison with the pure MXene electrode. The greatly enhanced capacitive behavior is related to the structure of hybrid electrodes, where MoO_3_ nanobelts not only serve as the interspacers of MXene to accelerate the in-time ion intercalation/extraction for fully expressing the MXene pseudocapacitance, but also are additional electrochemically active materials for the improvement of whole capacitance. Electrochemical impedance spectroscopy (EIS) was further performed to study understand the kinetics of electrode processes. As shown in Fig. 3f, all of Nyquist plots consist of a quasi-semicircle in the high-frequency regions and a nearly vertical line in the low-frequency regions. In the high-frequency section, the semicircle arc presents the charge transfer resistance (Rct) and electrode surface properties. According to the equal circuit fitting (Fig. 3f inset), the Rct value of pure MXene electrode is 2.2 Ω, while the hybrid electrodes show less diameter of semicircle arc which is 1.6, 1.8 and 2.0 Ω corresponding to the M/MoO_3_-10%, M/MoO_3_-20% and M/MoO_3_-30%, respectively. This might be due to that ionic conductivity of M/MoO_3_ electrodes is improved after embedding MoO_3_ nanobelts for fast charge transfer.

In the aspect of volumetric performance, as shown in Fig. 3g, M/MoO_3_-20% hybrid electrode yields the highest capacitance. The highest volumetric capacitance can reach 1817 F cm^−3^ at 3 mV s^−1^, much better than that of pure MXene (1225 F cm^−3^), approximately 1.5 times enhancement. Even at a high scan rate of 200 mV s^−1^, the volumetric capacitance can maintain 773 F cm^−3^ in sharp comparison with that of pure MXene electrode (459 F cm^−3^). Nevertheless, the M/MoO_3_-30% electrode displays reduced volumetric capacitance than that of M/MoO_3_-20% electrode, because the continuous addition of MoO_3_ will visibly augment the thickness of hybrid electrode inevitably (Fig. S3), detriment of not only the improvement of volumetric performance but also the deterioration of flexibility to some extent. In order to comprehensively understand the great enhancement of volumetric performance of M/MoO_3_-20% electrode, schematic diagram of the proposed synergistic effect is presented in Fig. 3h and could be explained from the following aspects. Firstly, according to the above electrochemical analysis, both of MXene and MoO_3_ show pseudocapacitive feature in acidic electrolyte in the potential of − 0.6–0.3 V. MXene can not only lower the intrinsic resistance of MoO_3_ nanobelts for fast electron transport in favor of achieving good rate capability, but also serve as the flexible substrate for MoO_3_ materials which cannot act as self-supporting film electrode directly without slurry mixing method or conductive substrate due to bad mechanical stability and conductivity. Given that MoO_3_ nanobelts distributed over the surface and interlamination of the MXene nanosheets, electrolyte ions are easily accessible to active surface including those of MoO_3_ nanobelts and MXene nanosheets, thereby contributing to high capacitive behavior. And the ultrathin feature of MoO_3_ nanobelts is beneficial for the improvement of volumetric performance in a certain range due to little increase in film thickness. But high mass percentage of MoO_3_ nanobelts will lead to the increase in the film thickness which is unfavorable to the continuous increase in the volumetric capacitance. Therefore, the high volumetric capacitance of optimal M/MoO_3_-20% electrode is from the good synergistic effect of the two materials. As a result, the outstanding volumetric feature of M/MoO_3_-20% electrode with excellent flexibility renders it most promising in achieving high volumetric energy density of flexible energy storage device.

**Fig. 4:**
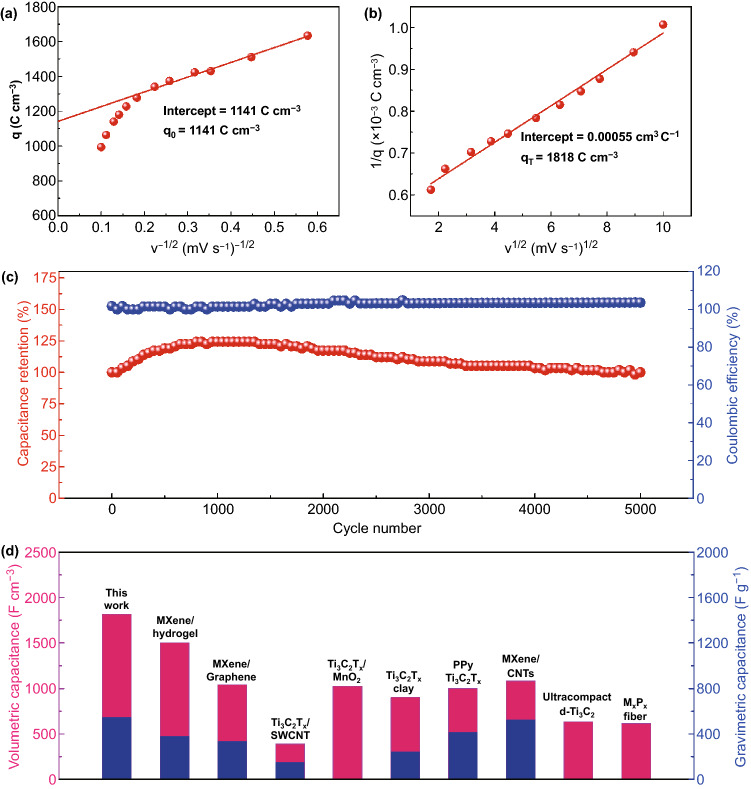
**a** Stored charges versus inverse of square root of the scan rates and **b** inverse of stored charges (*q*) versus the square root of the scan rates according to the data from M/MoO_3_-20% electrode, **c** cycling stability and Coulombic efficiency of the M/MoO_3_-20% electrode at a current density of 30 mA cm^−2^ for 5000 cycles, **d** comparison of capacitance between the prepared M/MoO_3_-20% electrode with previously reported state-of-the-art MXene-based electrodes

The electrochemical kinetics of M/MoO_3_-20% electrode was evaluated though Trasatti analysis method which is used to quantify the stored charges (*q*) during the energy storage process. The total amount of stored charge (9) consists of both outer (*q*_*o*_) and inner surface charges (*q*_*i*_), and they can be individually obtained by extrapolation of *q* to *v *= 0 and *v *→ ∞. The relevant formulas are as follows (Eqs. 9–11):

9 $$q_{T} = q_{i} + q_{0}$$

10 $$q = q_{\infty } + \kappa \upsilon^{ - 1/2}$$

11 $$\frac{1}{q} = \frac{1}{{q_{\infty } }} + \frac{{\upsilon^{1/2} }}{\kappa }$$

since the charge storage of the outer surface is a nondiffusion-controlled process, independent of scan rate, so *q*_*o*_ can be obtained from the extrapolation of *q* to *v *→ ∞ by using Eq. 10, where *q*_*o*_ is equaled to *q*_*∞*_, and the result of the linear fitting is shown in Fig. 4a. At the inner surface, the charge storage is opposite, controlled by ion diffusion. The total charge (*q*_*T*_) can be obtained from the extrapolation of *q* to *v* = 0 by Eq. 11 and the result of the linear fitting is shown in Fig. 4b. As a consequence, the outer and total charges of M/MoO_3_-20% electrode are calculated to be 1141 and 1818 C cm^−3^, respectively. At the scan rate of 3 mV s^−1^, the practical charge storage calculated is 1635 C cm^−3^, which accounts for 90% of the total charge storage (*q*_*T*_), indicating high electrochemical utilization of M/MoO_3_-20% electrode during the charge/discharge process. The result means that most of the active surfaces are accessible to electrolyte ions. Cycling stability is also an important factor for evaluating the performance of electrode materials for supercapacitors in practical applications. The cycling stability of the M/MoO_3_-20% electrode was conducted by using GCD at a current density of 30 mA cm^−2^ for 5000 cycles as provided in Fig. 4c. It can be seen that 100% of its initial specific capacitance is retained after continuous charging/discharging process, indicating good long-term cycle stability. This good stability might be associated with the tight laminar structure, where active materials are spatially defined in the interlayers for avoiding loss into the electrolyte. Comparison of the maximum volumetric capacitance and gravimetric capacitance of M/MoO_3_-20% electrode with other MXene-based state-of-the-art electrodes was made, which is depicted in Fig. 4d and Table S1. It is worth pointing out that, especially outstanding in terms of volumetric capacitance (1817 F cm^−3^ obtained in 1 M H_2_SO_4_ aqueous electrolyte), our electrode outperforms large majority of previously reported MXene-based flexible electrodes, such as MXene hydrogel (1500 F cm^−3^) [3], MXene/graphene (1040 F cm^−3^) [12], Ti_3_C_2_T_*x*_/SWCNT (390 F cm^−3^) [13], Ti_3_C_2_T_*x*_/MnO_2_ (1025 F cm^−3^) [18], Ti_3_C_2_T_*x*_ clay (900 F cm^−3^) [31], PPy/Ti_3_C_2_T_*x*_ (1000 F cm^−3^) [32], MXene/CNTs (1083 F cm^−3^) [33], Ultracompact d-Ti_3_C_2_ (633 F cm^−3^) [34] and M_X_P_X_ fiber (614.5 F cm^−3^) [35].

### Electrochemical Performance in a Symmetric Device

**Fig. 5:**
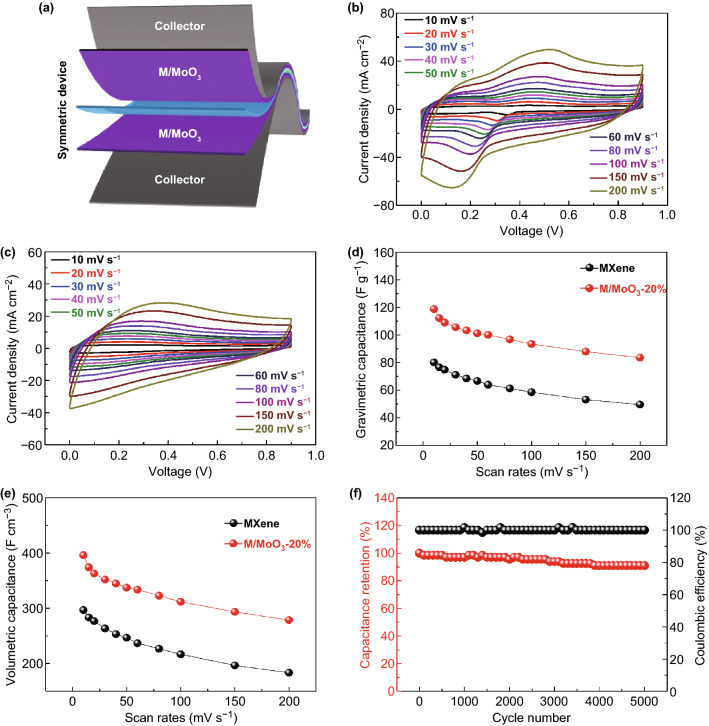
**a** Schematic representation of the assembled M/MoO_3_ symmetric device, **b** CV curves of the symmetric supercapacitor based on the M/MoO_3_-20% electrodes at various scan rates, **c** CV curves of the MXene symmetric supercapacitor at various scan rates, **d** gravimetric and **e** volumetric specific capacitance of the symmetric supercapacitor, **f** cycling stability and Coulombic efficiency of the symmetric supercapacitor at a current density of 30 mA cm^−2^ for 5000 cycles

In order to further evaluate the feasibility of the hybrid electrode in practical application for flexible energy storage devices, a M/MoO_3_ symmetric supercapacitor was fabricated by employing two pieces of identical M/MoO_3_-20% film electrode with a separator membrane in 1 M H_2_SO_4_ aqueous electrolyte, which is illustrated in Fig. 5a. The CV curves of the symmetric supercapacitor at different scan rates are given in Fig. 5b. It can be observed that all the CV curves exhibit a pair of redox peaks in the voltage range of 0–0.9 V at the scan rate varying from 10 to 200 mV s^−1^, demonstrating the predominant capacitance from redox pseudocapacitance. And the shape of redox peaks is still well maintained with a slight shift even when the scan rate reaches 200 mV s^−1^, indicating good rate capability. For MXene symmetric supercapacitor, the CV curves exhibit a pair of broader redox peaks at low scan rates (Fig. 5c). When the scan rates are increased to a high range, the redox peaks become more obscure especially in the discharging process. This indicates the M/MoO_3_ symmetric supercapacitor has different electrochemical processes compared with that of MXene symmetric supercapacitor. The specific capacitance of the symmetric device as a function of the scan rates is plotted in Fig. 5d, e based on the total active material. Notably, as expected, M/MoO_3_ symmetric supercapacitor exhibits an improved capacitive behavior. At the scan rate of 10 mV s^−1^, M/MoO_3_ symmetric supercapacitor has a capacitance of 396 F cm^−3^ (118.8 F g^−1^), while MXene symmetric supercapacitor only exhibits a capacitance of 297 F cm^−3^ (80.1 F g^−1^). With the scan rate increases to 200 mV s^−1^, a high capacitance retention of 70% is obtained for the M/MoO_3_ symmetric supercapacitor, higher than that of MXene symmetric supercapacitor (62%), reflecting a great boost in capacitance and a high capacitance retention. In addition, the cyclic stability of M/MoO_3_ symmetric supercapacitor was also tested with repeatedly being charged and discharged at a current density of 30 mA cm^−2^, as given in Fig. 5f. It shows that our device exhibits good cycling performance with a capacitance retention of 90% of the initial available specific capacitance after 5000 cycles.

**Fig. 6:**
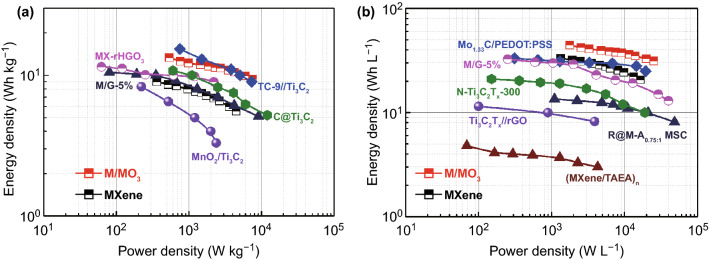
Ragone plots displaying energy and power densities of M/MoO_3_ symmetric supercapacitor in comparison with other state-of-the-art MXene-based supercapacitors

Benefiting from the high capacitance performance, the M/MoO_3_ symmetric supercapacitor delivers a maximum energy density of 13.4 Wh kg^−1^ at a power density of 534.6 W kg^−1^, much higher than that of MXene symmetric supercapacitor (9.0 Wh kg^−1^ at a power density of 360.5 W kg^−1^) and comparable with other reported symmetric supercapacitors such as M/G-5% (10.5 Wh kg^−1^) [12], MX-rHGO_3_ (11.5 Wh kg^−1^) [36], C@Ti_3_C_2_ (10.8 Wh kg^−1^) [37], MnO_2_/Ti_3_C_2_ (8.3 Wh kg^−1^) [38] and even asymmetric TC-9//Ti_3_C_2_ supercapacitor (15.4 Wh kg^−1^) [39], which is shown in Fig. 6. It is worth noting that the maximum volumetric energy density of M/MoO_3_ symmetric supercapacitor can reach 44.6 Wh L^−1^ at a power density of 1782 W L^−1^ in sharp with that of MXene symmetric supercapacitors (33.4 Wh L^−1^ at a power density of 1335 W L^−1^) and higher than those of previously reported state-of-the-art symmetric supercapacitors such as Mo_1.33_C MXene/PEDOT:PSS (33.2 Wh L^−1^) [40], Ti_3_C_2_T_*x*_/rGO-5 wt% (32.6 Wh L^−1^) [12], N–Ti_3_C_2_T_*x*_ − 300 (21 Wh L^−1^) [41], R@M-A_0.75:1_ MSC (13.5 Wh L^−1^) [42], MXene/rGO (8.6 Wh L^−1^) [43] and (MXene/TAEA)_*n*_ (5.1 Wh L^−1^) [22] (Table S2). Therefore, it is believed that this facile strategy by combining pseudocapacitive nanomaterials with MXene to improve the whole electrochemical performance and hold excellent flexibility of hybrid electrodes is considered be feasible for achieving high-energy-density flexible energy storage devices.

## Conclusions

Ultrathin MoO_3_ nanobelts and delaminated MXene nanosheets are integrated together by a facile and efficient vacuum-assisted method to fabricate all-pseudocapacitive and highly deformable M/MoO_3_ hybrid films. The excellent synergetic effect is achieved in acidic electrolyte where MXene nanosheets can express highest pseudocapacitance. In the hybrid structure, MoO_3_ nanobelts not only serve as the intercalators for the full advantage of MXene active surface but also provide additional pseudocapacitance for the whole high capacitance performance. Meanwhile, MXene is an excellent conductive material to lower the intrinsic resistance of MoO_3_ nanobelts for fast electron transport, thereby obtaining good rate capability. As a consequence, the as-prepared freestanding M/MoO_3_-20% hybrid film demonstrates an ultrahigh volumetric capacitance of 1817 F cm^−3^ (545 F g^−1^), almost 1.5 times higher than that of pure MXene film, and exceeds large majority of previously reported MXene-based flexible electrodes. Due to ultrathin feature of both MoO_3_ nanobelts and MXene nanosheets, outstanding flexibility is presented, which is bended, curled and even folded without cracks. Furthermore, the assembled symmetric supercapacitor device can obtain an excellent energy density of 44.6 Wh L^−1^ (13.4 Wh kg^−1^) at a power density of 1782 W L^−1^ in sharp with that of MXene symmetric supercapacitors (33.4 Wh L^−1^ at a power density of 1335 W L^−1^) in aqueous electrolyte. We believe that the work would facilitate the progress of MXene-based flexible electrodes in achieving high-energy-density energy storage devices.

## Electronic supplementary material

Below is the link to the electronic supplementary material.

Supplementary material 1 (PDF 651 kb)
