# Supplementary material for: Intercalating Ultrathin MoO3 Nanobelts into MXene Film with Ultrahigh Volumetric Capacitance and Excellent Deformation for High-Energy-Density Devices
Source: Nanomicro Lett. 2020 May 22;12:115. doi: 10.1007/s40820-020-00450-0 (PMC7770681; doi:10.1007/s40820-020-00450-0)
Supplement: Supplementary file 1 — Supplementary material 1 (PDF 651 kb) [file 40820_2020_450_MOESM1_ESM.pdf]

Supporting Information for

Intercalating Ultrathin MoO_3 Nanobelts into MXene Film with Ultrahigh Volumetric Capacitance and Excellent Deformation for High-Energy-Density Devices

Yuanming Wang[1,#], Xue Wang[1,#], Xiaolong Li[1], Rong Liu[2,*], Yang Bai[1], Huanhao Xiao[1], Yang Liu[1], Guohui Yuan[1,*]

[1]MIIT Key Laboratory of Critical Materials Technology for New Energy Conversion and Storage, School of Chemistry and Chemical Engineering, Harbin Institute of Technology, No. 92 West Dazhi Street, Harbin 150001, People's Republic of China

[2]Ocean College, Hebei Agricultural University, No. 52 Hebei Street, Qinhuangdao 066000, People's Republic of China

[#]Yuanming Wang and Xue Wang contributed equally to this work

*Corresponding authors. E-mail: yghhit@163.com (Guohui Yuan); 562204852@qq.com (Rong Liu)

Supplementary Figures

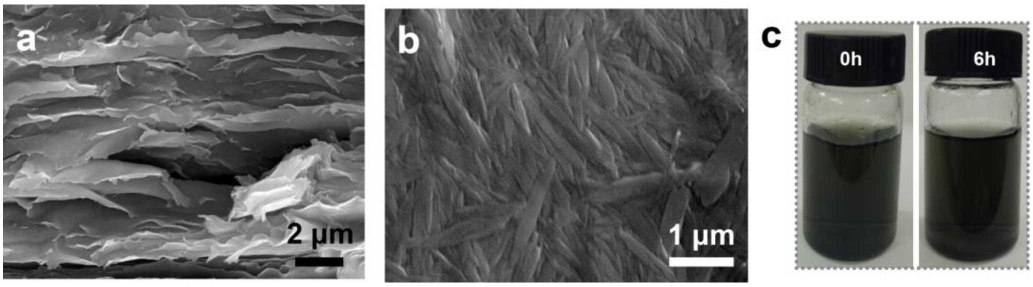

Fig. S1 SEM images of **(a)** MXene nanosheets, **(b)** MoO_3 nanobelts. **(c)** The mixture solution including MXene nanosheets and MoO_3 nanobelts without any precipitate after standing for several hours

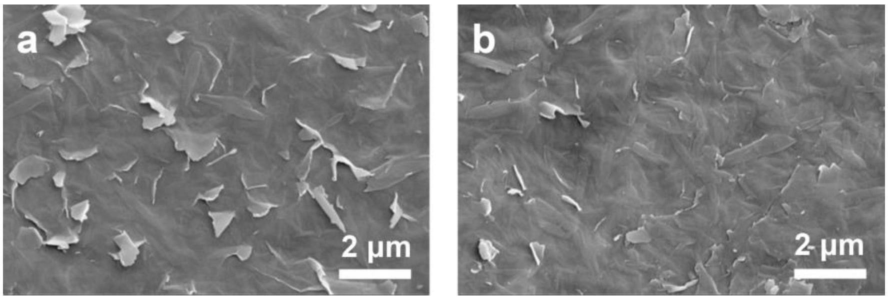

Fig. S2 Top-view SEM images of **(a)** M/MoO_3-10% and **(b)** M/MoO_3-30% hybrid films

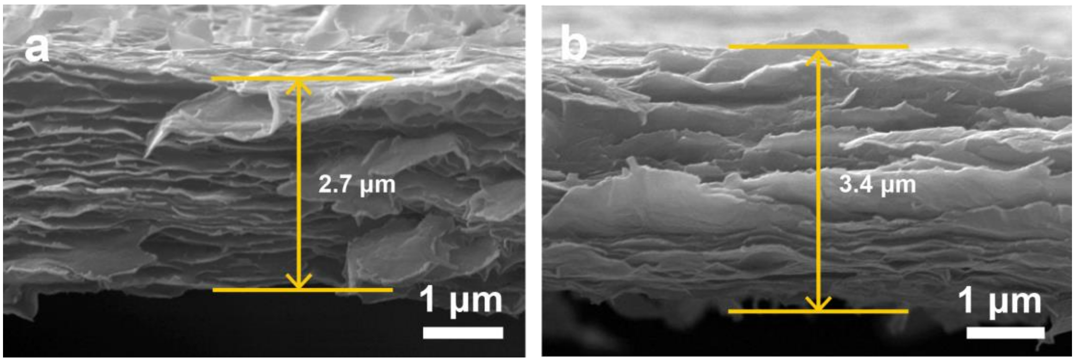

Fig. S3 Cross-section images of **(a)** pure MXene film and **(b)** M/MoO_3-30% hybrid films

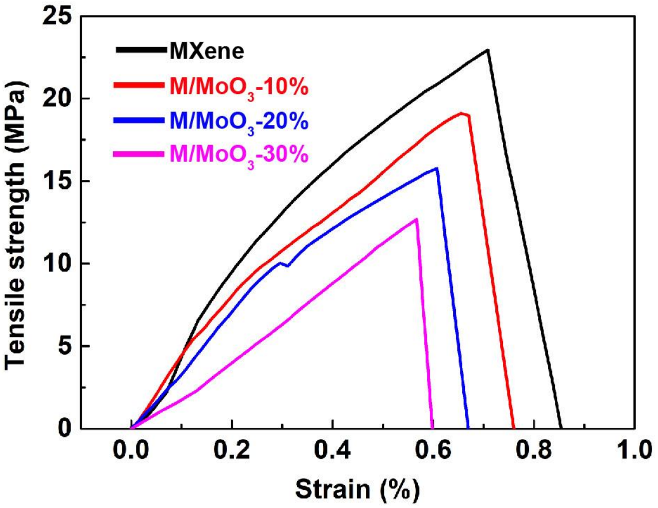

Fig. S4 Stress-strain curve of pure MXene film and hybrid films

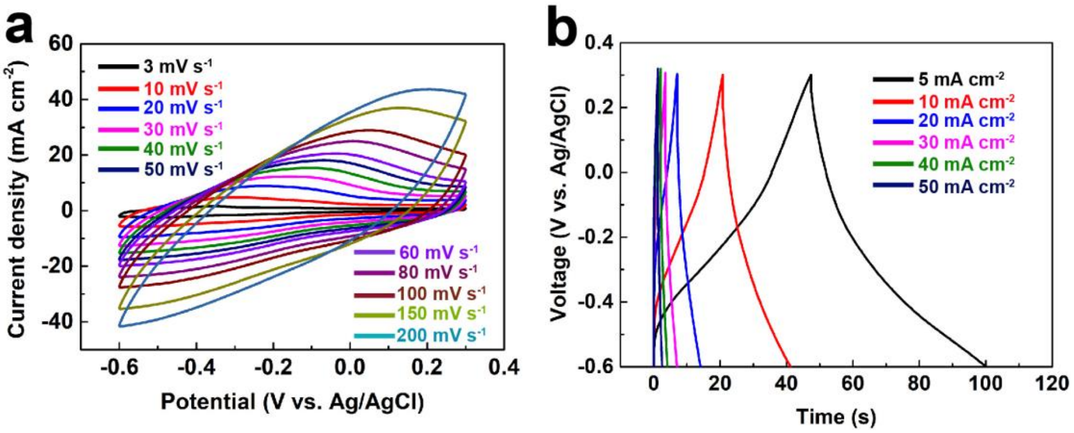

Fig. S5 (a) CV curves of the pure MXene electrode at various scan rates. **(b)** GCD profiles of pure MXene electrode at various current densities

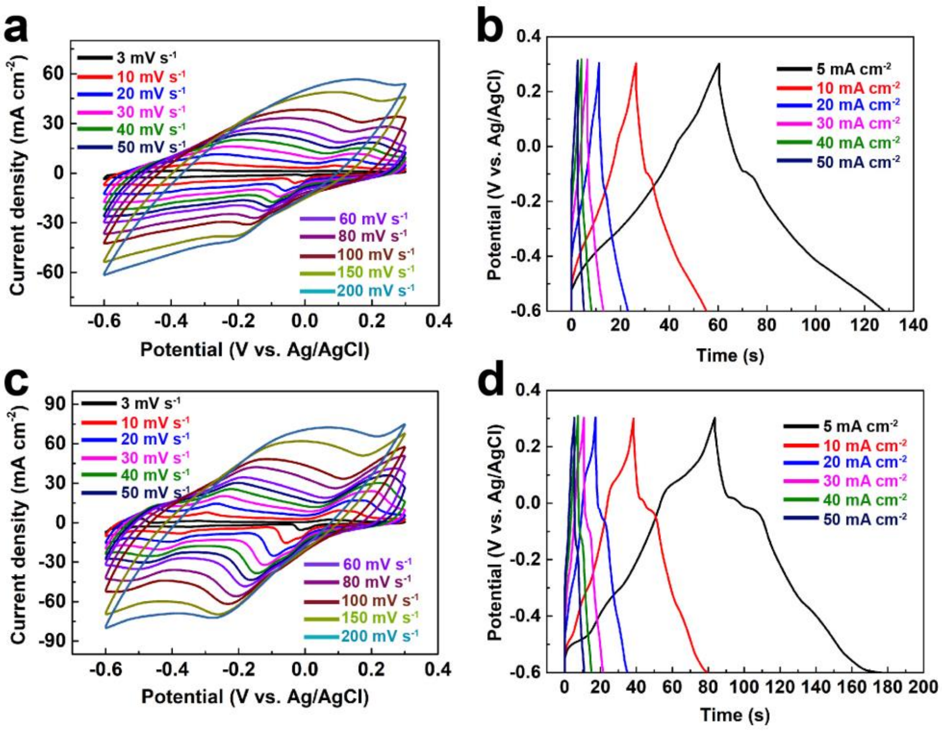

Fig. S6 (a) CV curves of the M/MoO_3-10% electrode at various scan rates. **(b)** GCD profiles of M/MoO_3-10% electrode at various current densities. **(c)** CV curves of the M/MoO_3-30% electrode at various scan rates. **(d)** GCD profiles of M/MoO_3-30% electrode at various current densities

Table S1 Comparison of the electrochemical performance of M/MoO_3-20% electrode with other MXene-based state-of-the-art electrodes

Materials	Electrolyte (mol L^{-1})	Potential (V)	Test condition	*C*g (F g^{-1})	*C*v (F cm^{-3})	Refs.
MXene hydrogel	3M H_2SO_4	-1.1--0.1	2 mV s^{-1}	380	1500	[S1]
$Ti_3C_2T_x$ clay	1M H_2SO_4	-0.35-0.2	2 mV s^{-1}	245	900	[S2]
MXene/Graphene	3M H_2SO_4	-0.7-0.3	2 mV s^{-1}	335.4	1040	[S3]
$Ti_3C_2T_x/MnO_2$	PVA/LiCl	0.8	1 A cm^{-3}	--	1025	[S4]
PPy/$Ti_3C_2T_x$	1M H_2SO_4	-0.2-0.35	5 mV s^{-1}	416	1000	[S5]
MXene/CNTs	3M H_2SO_4	-0.55-0.1	2 mA cm^{-2}	523	1083	[S6]
Ultracompact d- Ti_3C_2	1M Li_2SO_4	1V	2 mV s^{-1}	--	633	[S7]
$Ti_3C_2T_x$/SWCNT	1M $MgSO_4$	-0.8-0.1	2 mV s^{-1}	150	390	[S8]
M_XP_X fiber	PVA/ H_2SO_4	-0.65-0.2	5 mV s^{-1}	--	614.5	[S9]
MXene/MPFs	0.5M H_2SO_4	-0.3-0.3	1 A cm^{-3}	--	694.2	[S10]
**M/MoO_3-20%**	**1M H_2SO_4**	**-0.6-0.3**	**3 mV s^{-1}**	**545**	**1817**	**This work**

Table S2 Comparison of the electrochemical performance of M/MoO_3 hybrid electrode with other reported electrode materials for symmetric supercapacitors

Materials	Electrolyte (mol L^{-1})	C_V (F cm^{-3})	E_v (Wh L^{-1})	p_v (W L^{-1})	Refs.
$Ti_3C_2T_x$/rGO-5 wt%	3 M H_2SO_4	--	32.6	74400	[S3]
d-Ti_3C_2	Organic electrolyte	--	41	--	[S7]
$Mo_{1.33}C$ MXene /PEDOT:PSS	PVA/H_2SO_4 gel	568	33.2	19470	[S11]
N-$Ti_3C_2T_x$ -300	3 M H_2SO_4	--	21	18300	[S12]
R@M-$A_{0.75:1}$ MSC	PVA-KOH	267.9	13.5	48500	[S13]
MXene/rGO	PVA/ H_2SO_4	80	8.6	--	[S14]
$(MXene/TAEA)_n$	PVA/ H_2SO_4	--	5.1	4400	[S15]
PPy/l-Ti_3C_2	PVA-H_2SO_4	--	10	4000	[S16]
EG/MXene 1:3	PVA/H_3PO_4	216	3.4	1600	[S17]
MnO_x-Ti_3C_2 film	1 M Li_2SO_4	--	13.64	3755.61	[S18]
MXene/rGO fiber	PVA/H_2SO_4	256	5.1	1700	[S19]
MXene-MoS_2	PVA/LiCl gel	173.6	15.5	970	[S20]
**M/MoO_3-20%**	**1 M H_2SO_4**	**396**	**44.6**	**25080**	**This work**

Supplementary References

[S1] M. R. Lukatskaya, S. Kota, Z. Lin, M.-Q. Zhao, N. Shpige et al., Ultra-high-rate pseudocapacitive energy storage in two-dimensional transition metal carbides. Nat. Energy **2**(8), 17105 (2017). https://doi.org/10.1038/nenergy.2017.105

[S2] M. Ghidiu, M. R. Lukatskaya, M. Q. Zhao, Y. Gogotsi, M. W. Barsoum, Conductive two-dimensional titanium carbide 'clay' with high volumetric capacitance. Nature **516**(7529), 78-81 (2014). https://doi.org/10.1038/nature13970

[S3] J. Yan, C. E. Ren, K. Maleski, C. B. Hatter, B. Anasori et al., Flexible MXene/graphene films for ultrafast supercapacitors with outstanding volumetric capacitance. Adv. Funct. Mater. **27**(30), 1701264 (2017). https://doi.org/10.1002/adfm.201701264

[S4] J. Zhou, J. Yu, L. Shi, Z. Wang, H. Liu et al., A conductive and highly deformable all-pseudocapacitive composite paper as supercapacitor electrode with improved areal and volumetric capacitance. Small. **14**(51), 1803786 (2018). https://doi.org/10.1002/smll.201803786

[S5] M. Boota, B. Anasori, C. Voigt, M. Q. Zhao, M. W. Barsoum, Y. Gogotsi, Pseudocapacitive electrodes produced by oxidant-free polymerization of pyrrole between the layers of 2D titanium carbide (MXene). Adv. Mater. **28**(7), 1517-1522 (2016). https://doi.org/10.1002/adma.201504705

[S6] Z. Wang, S. Qin, S. Seyedin, J. Zhang, J. Wang et al., High-performance biscrolled MXene/carbon nanotube yarn supercapacitors. Small **14**(37), 1802225 (2018). https://doi.org/10.1002/smll.201802225

[S7] C. Yang, Y. Tang, Y. Tian, Y. Luo, Y. He, X. Yin, W. Que, Achieving of flexible, free-standing, ultracompact delaminated titanium carbide films for high volumetric performance and heat-resistant symmetric supercapacitors. Adv. Funct. Mater. **28**(15), 1705487 (2018). https://doi.org/10.1002/adfm.201705487

[S8] M. Q. Zhao, C. E. Ren, Z. Ling, M. R. Lukatskaya, C. Zhang et al., Flexible MXene/carbon nanotube composite paper with high volumetric capacitance. Adv. Mater. **27**(2), 339-345 (2015). https://doi.org/10.1002/adma.201404140

[S9] J. Zhang, S. Seyedin, S. Qin, Z. Wang, S. Moradi et al., Highly conductive $Ti_3C_2T_x$ MXene hybrid fibers for flexible and elastic fiber-shaped supercapacitors. Small **15**(8), 1804732 (2019). https://doi.org/10.1002/smll.201804732

[S10] W. Zhao, J. Peng, W. Wang, B. Jin, T. Chen et al., Interlayer hydrogen-bonded metal porphyrin frameworks/MXene hybrid film with high capacitance for flexible all-solid-state supercapacitors. Small **15**(18), 1901351 (2019). https://doi.org/10.1002/smll.201901351

[S11] L. Qin, Q. Tao, A. El Ghazaly, J. Fernandez-Rodriguez, P. O. Å. Persson, J. Rosen, F. Zhang, High-performance ultrathin flexible solid-state supercapacitors based on solution processable $Mo_{1.33}C$ MXene and PEDOT:PSS. Adv. Funct. Mater. **28**(2), 1703808 (2018). https://doi.org/10.1002/adfm.201703808

[S12] Y. Tian, W. Que, Y. Luo, C. Yang, X. Yin, L. B. Kong.,Surface nitrogen-modified 2D titanium carbide (MXene) with high energy density for aqueous supercapacitor applications. J. Mater. Chem. A **7**(10), 5416-5425 (2019). https://doi.org/10.1039/c9ta00076c

[S13] H. Li, X. Li, J. Liang, Y. Chen, Hydrous RuO_2-decorated mxene coordinating with silver nanowire inks enabling fully printed micro-supercapacitors with extraordinary volumetric performance. Adv. Energy Mater. **9**(15), 1803987 (2019). https://doi.org/10.1002/aenm.201803987

[S14] C. Couly, M. Alhabeb, K. L. Van Aken, N. Kurra, L. Gomes et al., Asymmetric flexible MXene-reduced graphene oxide micro-supercapacitor. Adv. Electron. Mater. **4**(1), 1700339 (2018). https://doi.org/10.1002/aelm.201700339

[S15] W. Tian, A. VahidMohammadi, Z. Wang, L. Ouyang, M. Beidaghi, M. M. Hamedi, Layer-by-layer self-assembly of pillared two-dimensional multilayers. Nat. Commun. **10**(1), 2558 (2019). https://doi.org/10.1038/s41467-019-10631-0

[S16] M. Zhu, Y. Huang, Q. Deng, J. Zhou, Z. Pei et al., Highly flexible, freestanding supercapacitor electrode with enhanced performance obtained by hybridizing polypyrrole chains with MXene. Adv. Energy Mater. **6**(21), 1600969 (2016). https://doi.org/10.1002/aenm.201600969

[S17] H. Li, Y. Hou, F. Wang, M. R. Lohe, X. Zhuang, L. Niu, X. Feng, Flexible all-solid-state supercapacitors with high volumetric capacitances boosted by solution processable MXene and electrochemically exfoliated graphene. Adv. Energy Mater. **7**(4), 1601847 (2017). https://doi.org/10.1002/aenm.201601847

[S18] Y. Tian, C. Yang, W. Que, X. Liu, X. Yin, L. B. Kong, Flexible and free-standing 2D titanium carbide film decorated with manganese oxide nanoparticles as a high volumetric capacity electrode for supercapacitor. J. Power Sources **359**(332-339 (2017). https://doi.org/10.1016/j.jpowsour.2017.05.081

[S19] S. Seyedin, E. R. S. Yanza, Joselito M. Razal, Knittable energy storing fiber with high volumetric performance made from predominantly MXene nanosheets. J. Mater. Chem. A **5**(46), 24076-24082 (2017). https://doi.org/10.1039/c7ta08355f

[S20] X. Chen, S. Wang, J. Shi, X. Du, Q. Cheng et al., Direct laser etching free-standing MXene-MoS_2 film for highly flexible micro-supercapacitor. Adv. Mater. Interfaces **6**(22), 1901160 (2019). https://doi.org/10.1002/admi.201901160
